# Dissecting the Landscape of Activated CMV-Stimulated CD4+ T Cells in Humans by Linking Single-Cell RNA-Seq With T-Cell Receptor Sequencing

**DOI:** 10.3389/fimmu.2021.779961

**Published:** 2021-12-07

**Authors:** Menghua Lyu, Shiyu Wang, Kai Gao, Longlong Wang, Xijun Zhu, Ya Liu, Meiniang Wang, Xiao Liu, Bin Li, Lei Tian

**Affiliations:** ^1^ College of Life Sciences, University of Chinese Academy of Sciences, Beijing, China; ^2^ BGI-Shenzhen, Shenzhen, China; ^3^ Tsinghua Shenzhen International Graduate School, Tsinghua University, Shenzhen, China; ^4^ Shanghai Institute of Immunology, Shanghai JiaoTong University School of Medicine, Shanghai, China; ^5^ Department of Neurology, Shenzhen People’s Hospital (The First Affiliated Hospital of Southern University of Science and Technology, The Second Clinical Medical College of Jinan University), Shenzhen, China

**Keywords:** CMV pp65, single-cell mRNA-seq, paired TCR-seq, CD4+ T cells, CD4+ CTL, Treg

## Abstract

CD4+ T cells are crucial in cytomegalovirus (CMV) infection, but their role in infection remains unclear. The heterogeneity and potential functions of CMVpp65-reactivated CD4+ T cell subsets isolated from human peripheral blood, as well as their potential interactions, were analyzed by single-cell RNA-seq and T cell receptor (TCR) sequencing. Tregs comprised the largest population of these reactivated cells, and analysis of Treg gene expression showed transcripts associated with both inflammatory and inhibitory functions. The detailed phenotypes of CMV-reactivated CD4+ cytotoxic T1 (CD4+ CTL1), CD4+ cytotoxic T2 (CD4+ CTL2), and recently activated CD4+ T (Tra) cells were analyzed in single cells. Assessment of the TCR repertoire of CMV-reactivated CD4+ T cells confirmed the clonal expansion of stimulated CD4+ CTL1 and CD4+ CTL2 cells, which share a large number of TCR repertoires. This study provides clues for resolving the functions of CD4+ T cell subsets and their interactions during CMV infection. The specific cell groups defined in this study can provide resources for understanding T cell responses to CMV infection.

## Introduction

Infections with cytomegaloviruses (CMV) and human herpesvirus 5 (HHV-5) are endemic in humans. Most immunocompetent CMV hosts show few or no clinical symptoms in response to primary infection or during persistent infection. Although CMV infection is asymptomatic, the virus hijacks the resources of the host immune system throughout the latter’s lifespan by remaining latent and occasionally reactivating. Over time, CMV-responsive T-cells constitute an average of 10% of the entire T-cell repertoire of the host (1), having deleterious effects on immune senescence and health outcomes in the elderly (2). In addition, CMV infection can have devastating consequences in immunocompromised populations, including fetuses and patients undergoing transplantation.

Reconstruction of CMV-specific T cells has emerged as an effective method of reducing CMV infection and reactivation in immunocompromised individuals. Data from patients who have undergone hematopoietic stem cell transplantation (HSCT) have shown that recovery from CMV-induced diseases correlates with the reconstruction of CMV-specific CD4+ and CD8+ T-cell pools (3–5), with the recovery of CD4+ T cells regarded as a prerequisite (6). CMV-specific CD4+ T cells are thought to stimulate the expansion of CMV-specific CD8+ T cells, resulting in a more effective clearance of virus from serum than treatment with CD8+ T cells alone (7). Furthermore, infusion of CD4+ T cells into immunocompromised mice was found to effectively repress CMV reactivation, further suggesting a pivotal role of CD4+ T cells in anti-CMV immunity. However, CD4+ T cells are heterogeneous, and their composition, function, and interaction in anti-CMV immunity remain unclear, precluding adoptive immune therapy in CMV-infected individuals.

Studies evaluating the roles of CMV-specific CD4+ T cell subsets in anti-CMV immunity have revealed that CD4+ cytolytic T cells (CD4+ CTL), regulatory T cells (Tregs), and CD4+ memory T cells are involved in immune responses to CMV infection in humans, nonhuman primates, and rodents. CD4+ CTLs were first identified in chronic viral infections, such as with lymphocytic choriomeningitis virus (LCMV), hepatitis B virus (HBV), and CMV. These cells show strong antiviral effects in anti-CMV immunity through their helper functions and induction of cytotoxicity. CD4+ CTLs manifest helper functions through their expression of cytokines and chemokines, such as IFN-γ and TNF-α (8), which promote the activation of CD8+ T cells; recruit innate immune cells, including natural killer (NK) cells and monocytes, to inflammatory sites, and directly inhibit virus replication (9). CD4+ CTLs manifest cytotoxicity through the Fas/FasL pathway, mediating the death of infected B cells presenting viral epitopes with major histocompatibility complex class II (MHC-II) molecules (10, 11). CD4+ CTL also manifest cytotoxicity through the perforin–granzyme pathway (12), based on the CTL recognition of target cells in an MHC-II-dependent manner (13), when MHC-II is upregulated in epithelial cells following CMV infection. Despite advances in understanding the functions of CD4+ CTLs in CMV infection, the derivation of these cells remains unclear. Based on findings in other infectious diseases, CD4+ CTLs are thought to originate from effector cells (14, 15). Recent evidence from studies on transcriptome factors has suggested that these cells can also directly differentiate from activated naïve cells (16–18).

The functions of Treg cells during CMV infection are also unclear. *Ex vivo* stimulation of human Treg cells from CMV-seropositive individuals with CMV was shown to attenuate the proliferation of autologous CD8+ T cells and, to a lesser extent, other subsets of CD4+ T cells through the PD-1 pathway (19). However, CMV reactivation following HSCT did not correlate with the numerical reconstruction of CD4+CD25highCD127- Tregs, and conventional T cells in these patients expressed high levels of the proliferation marker Ki67 indicating that their activation and proliferation were not obstructed by Tregs (20). Selectively deleting Tregs in animal models is a classical method to verify Treg function in infectious situations (21) and has been used to evaluate the negative regulatory function of Tregs in some antiviral immunities. However, deleting Tregs could not determine their function in CMV infection. In mice, the deletion of Treg cells decreased murine cytomegalovirus (MCMV) reactivation in the spleen but enhanced its activation in the salivary glands (22).

CD4+ T cells perform many essential functions, including stimulating B cells to mature and secrete antibodies and supporting cytotoxic CD8+ T cells and phagocytes to mount rapid and effective protection against infections (1). Despite their importance, technical limitations have often prevented the comprehensive analysis of CD4+ T cells. T-cell receptor (TCR) sequences are highly diverse, with an estimated tens of millions of unique TCR-expressing T-cell clones largely unique to individuals (23, 24), limiting the ability to directly compare the abundances of T-cell clones across multiple samples. Antigen-specific T cells can be isolated using peptide-MHC (pMHC) multimers (2), and this method has been used in the parallel detection of T cells on a large scale (3–7). This method, however, depends on advance knowledge of the relevant human leukocyte antigen (HLA) molecules and antigenic epitopes, which in most cases cannot be efficiently predicted (8). In addition, the process involved in generating pMHC multimers is complicated, and few usable pMHC II multimers are available for CD4+ T cells. Due to the variety of HLA alleles (11) and the complexity of many antigen genomes, it is difficult to thoroughly analyze antigen-specific T cells with limited numbers of pMHC multimers. Although the enzyme-linked immune absorbent spot (ELISpot) can also be used to analyze antigen-specific T cells, this method is limited to detecting a single/or a limited panel of cytokine(s) and is therefore not sufficiently comprehensive to analyze different T cell subtypes that are involved in the protection against pathogen infection.

These challenges may be overcome by enriching for T cells specific for CMVpp65 through the expression of the T cell activation marker CD154 induced by stimulation *in vitro*, combined with single-cell mRNA and paired VDJ sequencing to dissect the CD4+ T cell responses (25). This method of isolating CMV-specific CD4+ T cells has several advantages, in that it is HLA-independent, can capture activated CD4+ T cells of different phenotypes, and is useful for high-throughput analysis. Comprehensive analysis of CMV-reactivated CD4+ T cells showed that a large proportion of these cells were CMV-reactivated Treg cells, with a Th1 phenotype, as shown by expression of IFNG and TNF, enhanced migration ability, and multiple inhibitory functions. In addition, this study found that both CD4+ CTL1 and CD4+ CTL2 have polyfunctional phenotypes, experienced clonal expansion, and had a large overlap in TCR repertoire. Furthermore, a group of recently activated CD4+ T cells (CD4+ Tra) cells were found to express cytolytic factor. These findings showed that CMV-reactivated CD4+ T cells were heterogeneous, consisted of a balance between CMV-specific Treg and effector T cells, and suggested that the composition of CD4+ T cells may be critical for adoptive T cell therapy in patients infected with CMV.

## Results

### CMV pp65-Specific CD4+ T Cells Have Typical Antiviral Profiles

**Figure 1:**
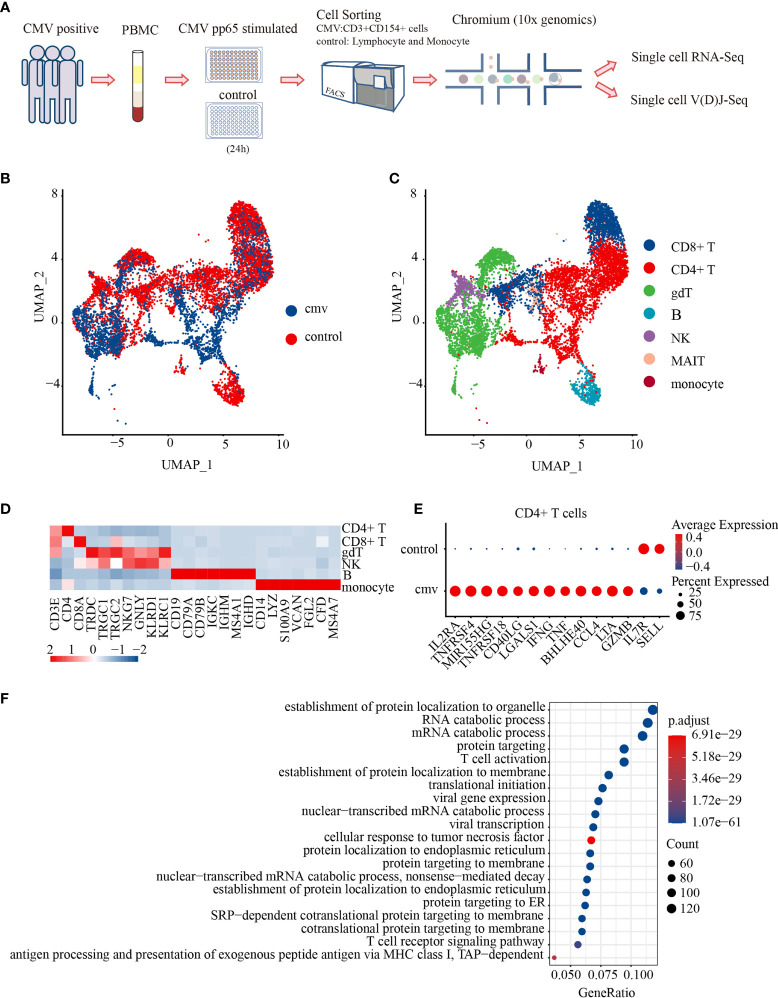
Characterization of the antiviral profiles of CMV pp65-specific CD4+ T cells. **(A)** Experimental workflow for single-cell analysis of CD4+ T cells from PBMC of three donors. Cells stimulated *in vitro* with CMV pp65 were cultured and sorted, with reactivated CMV-stimulated T cells gated for CD3+CD154+, and control monocytes and lymphocytes gated for FSC-SSC, followed by 5′ single-cell RNA and paired T-cell receptor sequencing. UMAP embeddings of merged scRNA-seq profiles from control and CMV-stimulated immune cells were plotted and colored by **(B)** sample and **(C)** cell cluster. **(D)** Heat map of scaled mean gene expression of the major canonical markers (columns) detected in different cell types in merged CMV and control cells (rows). **(E)** Dot plot of differentially expressed genes (DEGs), showing both the levels of expression and the percentages of CD4+ T cells in CMV and control samples. **(F)** Gene Ontology (GO) analysis of DEGs in CMV and control CD4+ T cell samples. The Top 20 enriched GO terms are ordered on the y-axis. The x-axis represents the gene percentage in enriched GO terms. The sizes of the dots represent the number of genes included in each GO term. The color gradient of dots represents the adjusted p-values of each enriched GO term.

Circulating antigen-specific T cells are rare in peripheral blood during the latent stage of CMV infection, representing 0.5% to 4% of the CD8+ T-cell pool and 0.05% to 1.6% of the CD4+T cell pool (26). To isolate CMV-specific CD4+ T cells, peripheral blood mononuclear cells (PBMCs) were cultured in the presence or absence of CMV-pp65 peptides for 24 h (25, 27–29). CMV-reactivated CD4+ T cells from three donors were sorted and pooled together for single-cell mRNA-seq and paired VDJ-seq using the 10 × Chromium platform. Single control cells were acquired from each donor by lymphocyte and monocyte sorting with forward scatter and side scatter (FSC/SSC) parameters; the sorted cells were also mixed and subjected to single-cell sequencing (**Figure 1A**). Flow cytometry analysis (**Supplemental Figure 1A**) showed that the expression of CD154 was much higher in CMV-stimulated than in control CD4+ T cells (**Supplemental Table 1**).

After stringent quality control and filtering using multiple criteria, RNA-seq data were obtained from 2,847 and 6,493 single cells from the CMV and control libraries, respectively. These analyses detected a mean of 3,041 genes per CMV infected cell and 1,947 genes per control cell. Productive VDJ sequences were obtained for 1,271 CMV cells and 3,557 control cells. The cells of the three donors from the CMV-infected and control cells were subsequently integrated for further analysis. The unsupervised clustering of all cells in the integrated data resulted in 15 distinct clusters: CD8+ T, γδT, B, NK, mucosal-associated invariant T (MAIT), monocytes, and nine clusters of CD4+ T cells (**Figures 1B–D**). We first showed CD4+ T cells as one cluster to analyze their shared characters and to be able to make comparison with previous studies.

To reveal the potential function of CMV-stimulated CD4+ T cells, CMV and control CD4+ T cells with mRNA and/or productive VDJ data (CMV: 1,200 cells, control: 1,911 cells) were selected for further analysis. Both mRNA and VDJ information was available for 974 cells in the CMV and 1,648 in the control group (**Supplemental Table 2**). Genes differentially expressed by these CMV and control CD4+ T cells were analyzed. CMV CD4+ T cells showed a typical T cell activation profile, including increased expression of *IL2RA*, *TNFRSF4*(*OX40)*, *MIR155HG*, *TNFRSF18*, *CD40LG*, and *LGALS1* and decreased expression of *IL7R* and *SELL*. These cells also express genes encoding the inflammatory cytokines *IFNG* and *TNF* (30, 31), the T-bet-independent IFN-γ production inducer *BHLHE40* (32), the pro-inflammatory chemokine *CCL4*, and the cytotoxic molecules *LTA* and *GZMB* (**Figure 1E**). These results suggest that CMV CD4+ T cells consist of several groups of activated multiple-cytokine-producing antiviral cells. These results were further confirmed by Gene Ontology (GO) analysis, which showed that differentially expressed genes (DEGs) were significantly enriched in pathways such as T cell activation and cellular response to tumor necrosis factors (**Figure 1F**). Consistent with previous reports using CD154 as a marker for antigen-specific CD4+ T cells (25), the cells obtained here with the same strategy exhibited a typical activated anti-viral response.

### Polyfunctionality Profiles of CMV pp65-Specific CD4+ T Cell Subsets

**Figure 2:**
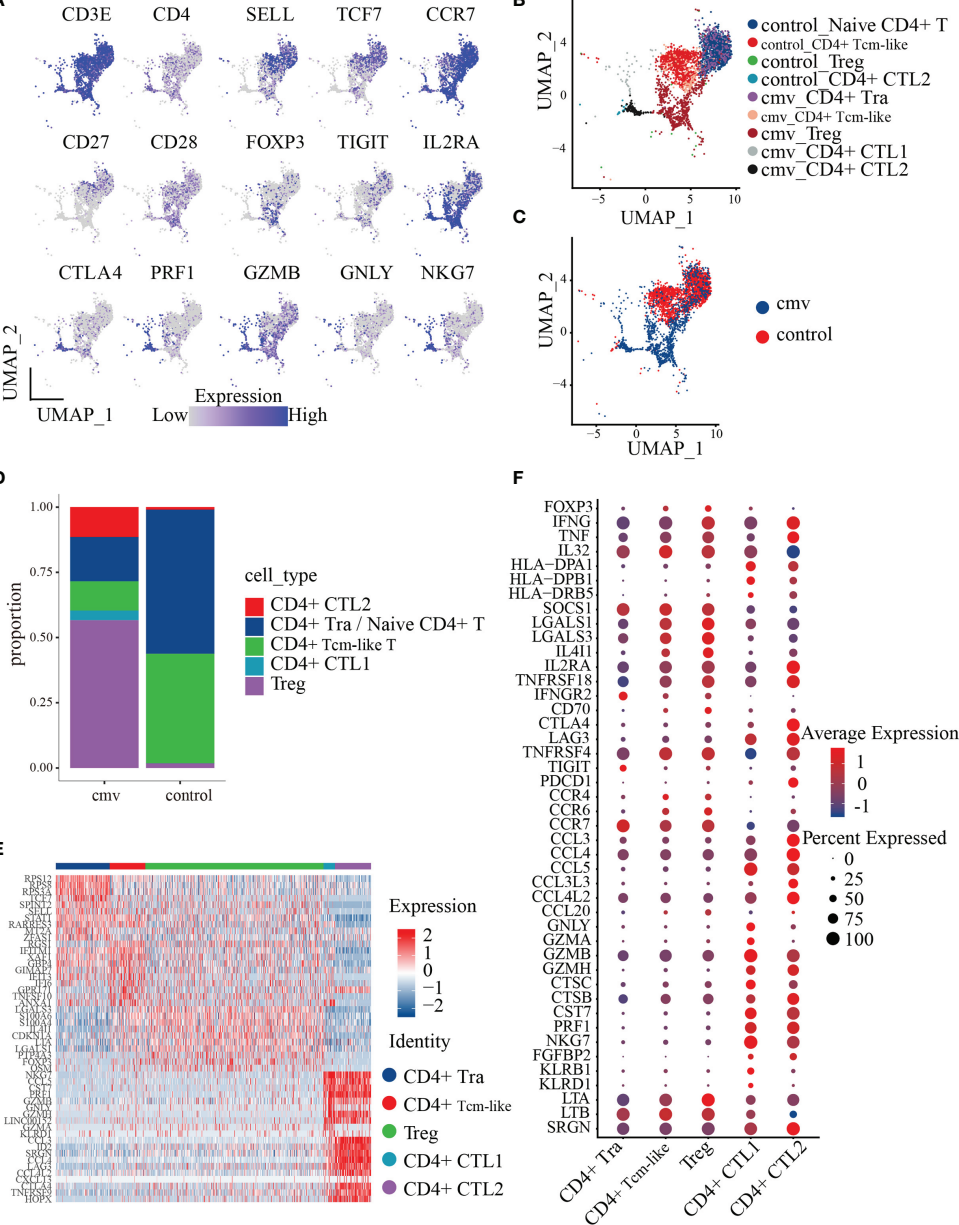
Polyfunctionality profiles of CMV pp65-stimulated CD4+ T cell subsets. **(A)** UMAP projections for the merged CD4+ T cells colored by expression of the naïve CD4+ T/Tcm-like cell markers *CD3E*, *CD4*, *SELL*, *TCF7*, *CCR7*, *CD27*, and *CD28*; Treg markers *FOXP3*, *IL2RA*, and *TIGIT*; the cytotoxicity markers *GZMB*, *NKG7*, and *PRF1*. Relative expression was normalized across CMV and control datasets. **(B, C)** UMAP embeddings of merged scRNA-seq profiles from control and stimulated (CMV) CD4+ T cells plotted and colored by cell cluster **(B)** and sample **(C)**. Subpopulations of CD4+ T cells colored in **(B)** were identified by the canonical markers described in **Table 2**. **(D)** Distribution of the abundance of the subsets of CMV and control CD4+ T cells. **(E)** Heat map of the five subsets of CMV CD4+ T cell cells with the Top10 DEGs between each pair. **(F)** Dot plot of highly featured genes expressed in the five CD4+ T cell subsets in CMV.

To date, nine CD4+T cell subtypes have been described (**Figure 2B**), based on markers from our previous study (33) and the Human Cell Atlas (34, 35). Control CD4+ T cells consisted of four clusters: naïve CD4+ T cells/CD4+ central memory like T (Tcm-like) cells expressing *CCR7*, *SELL*, and *TCF7*; CD4+ cytotoxic T2 cells (CD4+ CTL2) expressing *GZMB*, *NKG7*, and *PRF1*; and a Treg cluster expressing Foxp3 and IL2RA. CMV-stimulated CD4+ T cells consisted of five clusters: recently activated CD4+ T (Tra) cells/CD4+ Tcm-like cells expressing CD154 and naïve markers (*CCR7*, *SELL*, and *TCF7*); two cytotoxic T cell clusters (CD4+ CTL1 and CD4+ CTL2) expressing *GZMB*, *NKG7*, and *PRF1* and distinguished by different expressions of chemokines (CD4+ CTL1 highly expressed *CCL5*, CD4+ CTL1 highly expressed *CCL3* and *CCL4*); a Treg cluster expressing *Foxp3* and *IL2RA* (**Table 1** and **Figures 2A–C**); and CD4+ central memory-like T cells and CD4+ naïve T cells which were further discriminated by GSEA analysis, as DEGs between CD4+ naïve T and CD4+ Tcm-like cells significantly enriched in the gene sets such as “GSE11057 NAÏVE VS MEMORY CD4 TCELL DN” and “GSE11057 NAÏVE VS CENT MEMORY CD4 TCELL DN” (**Supplemental Table 3**). The proportions of each subtype are shown in **Figure 2D**. The ratio of naïve to memory control CD4+ T cells was consistent with previous fluorescence-activated cell sorting (FACS) data (36). To attribute cells to their corresponding donor, PBMCs from the three donors were subject to bulk RNA-seq for subsequent single-nucleotide polymorphism (SNP) identification, and the identity of each cell was determined based on these natural genetic variations (37). Cells from donor 1 and donor 2 were generally similar (**Supplemental Figures 2A–C** **)**. Few cells were obtained from donor 3, with this donor accounting for 1.58% of the total CD4+T cells from the three donors. These results showed that CMV-stimulated CD4+ T cells were highly enriched in Treg cells and CD4+ CTLs.

**Table 1:** Cell type markers.

Cell type	Markers
Naïve CD4+ T/Tcm-like	CD3E+, CD4+, SELL+, CD27+, TCF7+, CCR7+
CD8+ T	CD3E+, CD8A+, CD8B+, CD4-
γδT	CD3E+CD4-CD8B-CD8aa+/-, TRDC+, TRGC1+, TRGC2+
Treg	CD3E+, CD4+, FOXP3+, IL2RA+
Recently activated CD4+ T	CD3E+, CD4+, SELL+, TCF7+, CCR7+, CD154+
B	CD19+, CD79A+, CD79B+, MS4A1+, IGKC+, IGHM+
NK	CD3E-, NKG7, GNLY, NKG7, KLRD1, KLRC1
CD4+ CTL1	CD3E+, CD4+, CD27-, CD28-, GZMB+, NKG7+, PRF1+, CCL3+, CCL4+
CD4+ CTL2	CD3E+, CD4+, CD27-, CD28-, GZMB+, NKG7+, CCL5
Monocyte	LYZ+, S100A9+, CD14+, FGL2+, MS4A7+
MAIT	TRAV1-2/TRAJ33, TRAV1-2/TRAJ20, TRAV1-2/TRAJ12

To investigate the transcriptome features of the five CMV-stimulated CD4+ T cell subsets, CD4+ T cells from the CMV dataset (1,200 cells) were selected for further analysis. The five CD4+ T cell subsets were compared with each other using the *FindAllMarkers* function, with the resulting DEGs shown in **Supplemental Table 4**. The top 10 DEGs (sorted by the logFoldChange parameter) were found to differ from each other, indicating that these subsets may have distinct phenotypes (**Figure 2E**). The phenotype of each subset was therefore analyzed based on the top 10 DEGs and feature genes previously identified in these subsets.

To understand the phenotype and role of Treg cells during CMV infection, their gene expression profiles were analyzed. These cells are *FOXP3*+*IL2RA+TNFRSF4+*, as well as expressing proinflammatory factors such as *IFNG* and *TNF*. When compared with the four other CMV-stimulated CD4+ T subsets (i.e., CD4+ Tra cells, CD4+ Tcm-like cells, CD4+ CTL1, and CD4+ CTL2 cells), the Treg cells showed a significantly higher expression of the stable marker *SOCS1*, the cytotoxicity-related molecule *LTA*, and a series of proteins encoded by genes related to inhibition, such as *LGALS1* (38), *LGALS3* (39), and *IL4I1* (40) and the costimulatory molecule *CD70* (adjusted p < 0.01 each) (**Figure 2F**). The expression by Tregs of the chemokine receptors *CCR4*, *CCR6*, and *CCR7* indicate their chemotaxis toward *CCL3* and *CCL5*, the latter of which is highly expressed by CD4+ CTL1 and CD4+ CTL2 cells, and the homing to secondary lymphoid organs. Moreover, the high level of expression of *CCL20*, which encodes a chemokine that binds to CCR6 in Tregs, suggests that these cells cluster in a self-sustaining positive feedback loop.

CD4+ CTLs play an important role in chronic antiviral responses and contribute directly to the containment of viral infection. Assessments of the phenotypes and functional mechanisms of the five CD4+ T subsets showed that both CD4+ CTL1 and CD4+ CTL2 expressed high levels of genes encoding cytotoxic molecules, including *GZMB*, *GZMH*, *CTSC*, *CTSB*, *CST7*, *PRF1*, *NKG7*, and *FGFBP2* (**Figure 2F**). The similar levels of expression of these cytotoxic markers in CD4+ CTL1 and CD4+ CTL2 indicate that they may employ the same mechanism of action, the granule exocytosis pathway, to initiate target cell apoptosis. This mechanism involves the regulated release of the contents of cytotoxic granules (e.g., PRF1, GZMB, GZMH, GZMA, CTSC, and GNLY) into the immunological synapses formed between effector and target cells, killing the latter (41). CD4+ CTL1 and CD4+ CTL2 also expressed high amounts of the chemokine CCL5 and the MHCII molecules HLA-DPA1 and HLA-DPB1, indicating that they may attract common targets to inflammatory sites and kill them in an MHC class II-dependent manner (13, 42). Besides, compared with CD4+ CTL2, CD4+ CTL1 expressed higher levels of many other cytotoxic molecules, such as GNLY, GZMA, KLRB1, and KLRD1 (**Figure 2F**), indicating that the functional spectrum of CD4+ CTL1 is wider than that of CD4+ CTL2. When compared with CD4+ CTL1, CD4+ CTL2 expressed higher levels of many genes encoding chemokines (such as *CCL3*, *CCL4*, *CCL3L3*, and *CCL4L2*, and co-stimulators, such as *CTLA4*, *LAG3*, *TNFRSF4*, and *PDCD1*), indicative of a terminal differentiation phenotype. These results suggest that CD4+ CTL2 may originate from CD4+ CTL1 cells, which is further supported by our TCR repertoire analysis.

**Figure 3:**
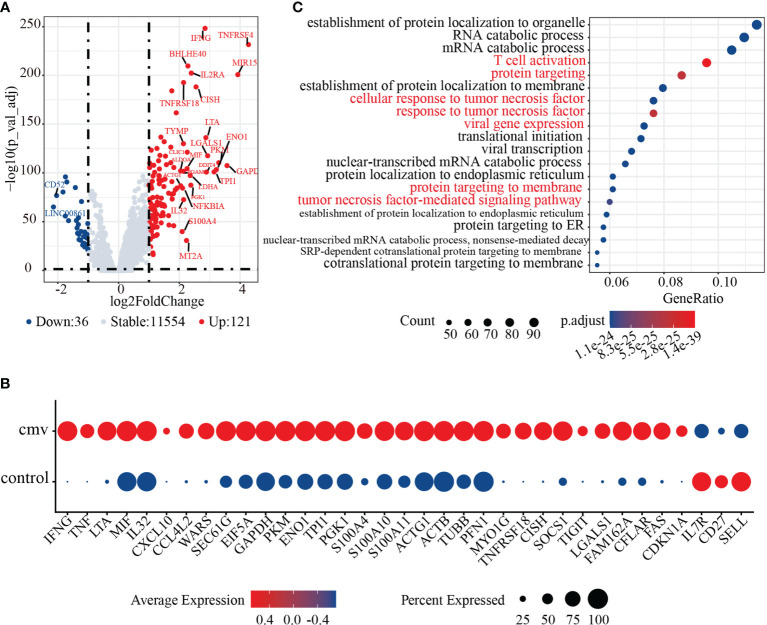
Activation characteristics of CMV pp65 stimulated recently activated CD4+ T (CD4+ Tra) cells. **(A)** Volcano plot showing the relationships between -log10(adjusted p value) (y-axis) and log2(fold change) (x-axis) for genes differentially expressed by CMV CD4+ Tra and control naive CD4+ T cells. Genes with log2 fold changes > 1 and adjusted p values < 0.05 were upregulated in CMV CD4+ Tra cells and highlighted in red, whereas genes with log2 fold changes < -1 and adjusted p values < 0.05 were downregulated in CMV CD4+ Tra cells and highlighted in blue. **(B)** GO analysis of DEGs by CMV CD4+ Tra cells and control naïve CD4+ T cells. The Top 20 enriched GO terms are ordered on the y-axis. The x-axis indicates gene percentages in enriched GO terms. The sizes of the dots represent the number of genes included in each GO term. The color gradient of dots represents the adjusted p-values for each enriched GO term. **(C)** Dot plot of highly featured genes expressed by CMV CD4+ Tra cells and control naïve CD4+ T cells.

CD4+ T cells recently activated by exposure to CMV pp65 peptides were found to cluster together with control naïve CD4+ T cells. Sorting of recently activated CD4+ T (Tra) cells by CD154 expression showed that these cells express high levels of genes encoding naïve T cell markers, such as *CCR7*, *TCF7*, and *SELL* (**Figures 2A–C**). To dissect the phenotype of the CD4+ T cells recently activated by CMV, we compared their gene expression with that of control naïve CD4+ T cells. In total, 981 genes were differentially expressed (adjusted p< 0.05) upon stimulation with the CMV pp65 peptides (**Figure 3A** and **Supplemental Table 5**). Of these, 121 genes were upregulated in CMV-activated cells and 36 were downregulated, with log2-fold changes > 1. These 121 upregulated genes included a group of genes encoding the cytokines and chemokines (*IFNG*, *TNF*, *LTA*, *MIF*, *IL32*, *CXCL10*, and *CCL4L2*), a group of genes regulating protein synthesis (e.g., *WARS*, *SEC61G*, and *EIF5A*), and a group involved in metabolism (43, 44) (e.g., *GAPDH*, *PKM*, *ENO1*, *TPI1*, and *PGK1*) (**Figure 3B**), findings indicative of cell activation (45). CD4+ Tra cells also expressed higher levels of S100 family genes encoding calcium-binding proteins (e.g., *S100A4*, *S100A10*, and *S100A11*) and cytoskeleton-related proteins (e.g., *ACTG1*, *ACTB*, *TUBB*, *PFN1*, and *MYO1G*), which had been reported increased in response to TCR engagement by antigen (46, 47). In addition, genes encoding many regulatory markers (e.g., *GITR* [*TNFRSF18*], *CISH*, *SOCS1*, and *TIGIT*) and cell apoptosis regulation markers (e.g., *LGALS1*, *FAM162A*, *CFLAR*, *FAS*, and *CDKN1A*) were strongly upregulated to maintain immune balance (48), although their expression levels differed in cells at different stages of differentiation (49, 50). The 36 downregulated genes included *CD127 (IL7R)*, *CD27*, and *SELL*, consistent with previous studies of T cell activation (51). GO analysis of the DEGs in recently activated CMV pp65-stimulated CD4+ T cells and control naïve CD4+ T cells demonstrated the significant enhancement of expression of genes associated with T cell activation, protein targeting, cellular response to tumor necrosis factor, viral gene expression, protein targeting to membrane, and the tumor necrosis factor-mediated signaling pathway (**Figure 3C**). These findings suggested that these phenotypically naïve CMVpp65-stimulated cells are in a state of recent activation.

### CMV pp65-Specific CD4+ T Cell Receptor Repertoire Shows a Reduction in Clonal Diversity

**Figure 4:**
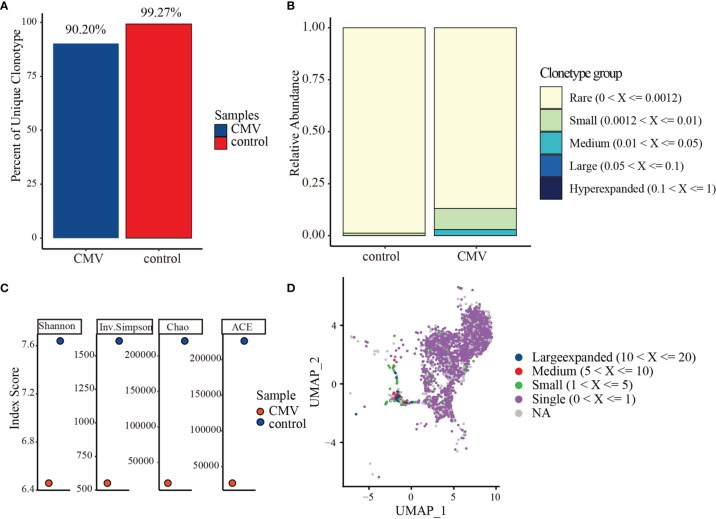
TCR repertoire analysis of CMV and control CD4+ T cells. **(A)** Percentages of unique (i.e., unexpanded) clonotypes of CMV and control CD4+ T cells. **(B)** Relative TCR repertoire abundance of CMV and control CD4+ T cells. **(C)** Diversity measures based on clonotypes by sample type using Shannon, Inverse Simpson, Chao, and abundance-based coverage estimator (ACE) indices. **(D)** Clonotype distributions of CD4+ T cells. Cloning frequencies ≤20 and > 10, ≤10 and >5, ≤5 and >1 were defined as large expanded, medium expanded, and small expanded, respectively.

The T-cell receptor (TCR) repertoire reflects the antigen specificity of T cells and their antigen experience in effector and memory subsets. Compared with the clonal diversity of the control CD4+ TCR repertoire, the clonal diversity of the CMV pp65-specific CD4+ TCR repertoire was reduced. Clones with the same VDJ (gene) and CDR3 nucleotide (nt) sequence were defined as being of the same clonotype (gene+nt), followed by a comparison of the features of the CD4+ TCR repertoire in CMV-stimulated and control cells. Analysis of the relative abundance of total CMV-stimulated and control CD4+T cells showed that the percentages of unique (i.e., unexpanded) clones in the CMV and control CD4+ T cells were 90.20% and 99.27%, respectively (**Figure 4A**). About 9.8% of the CMV-pp65-stimulated CD4+ T cells showed “medium” or “large” expansion (**Figure 4B**), indicating that they had undergone clonal amplification. Measured diversity using Shannon, Inverse Simpson, Chao, and abundance-based coverage estimator (ACE) across samples also showed an overall reduction in clonal diversity in the CMV sample (**Figure 4C**). To identify clones targeting the same antigens among cell subsets, the GLIPH2 algorithm (52) was utilized to cluster clones of CMV and control CD4+ T cells. The TCR convergence was found to be higher for CMV than for control CD4+ T cells (**Supplemental Tables 6** and **7**), with the TCR repertoire convergences being mainly between CD4+ CTL1 and CD4+ CTL2 in CMV. Consistent with the GLIPH2 result, combining VDJ sequences with transcriptome data (**Supplemental Table 8**) showed that the “larger” and “medium” expanded clones were mainly in the CD4+ CTL1 and CD4+ CTL2 subsets (**Figure 4D**).

### TCR Repertoire in CMV-Stimulated CD4+ T Cell Subgroups

**Figure 5:**
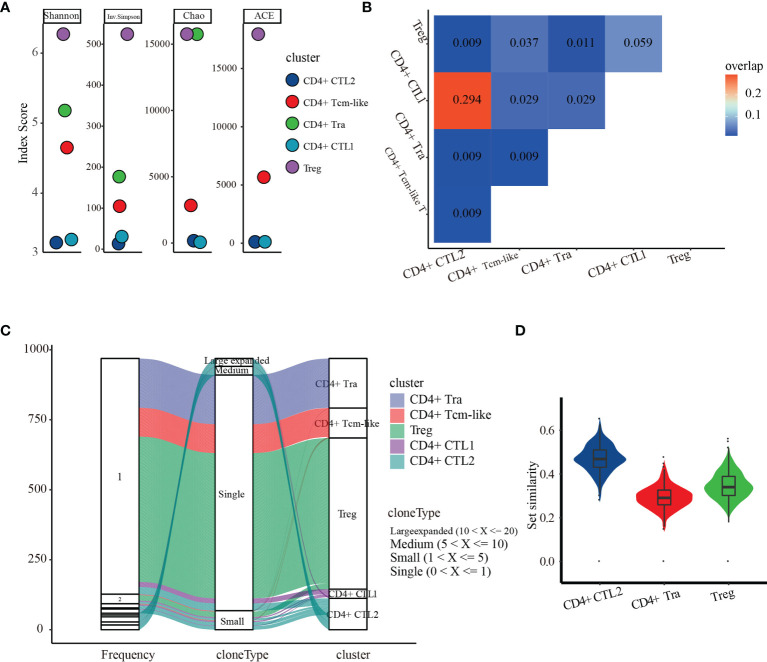
TCR repertoire analysis of the five CMV CD4+ T cell subsets. **(A)** Diversity measures based on clonotypes by cluster using Shannon, Inverse Simpson, Chao, and abundance-based coverage estimator (ACE) indices. **(B)** Clonal overlaps among the five CMV CD4+ T cell subsets. **(C)** Cloning frequency distribution in each subset. **(D)** Transcriptome similarity among CMV CD4+ CTLs, CD4+ Tra cells, and Treg cells.

To determine the dynamic changes in the CMVpp65-specific TCR repertoires of CD4+ T cell subsets, we analyzed the TCR repertoire of the five subgroups of CMV-stimulated CD4+ T cells. Measured TCR diversity using Shannon, Inverse Simpson, Chao, and ACE across these five cell clusters consistently showed reductions in clonal diversity in the order Treg, CD4+ Tra cells, CD4+ Tcm-like cells, CD4+ CTL1, and CD4+ CTL2 (**Figure 5A**). Calculation of the overlap in TCR repertoire among these clusters using overlap coefficient methods showed a large clonal overlap between CD4+ CTL1 and CD4+ CTL2 (**Figure 5B**); the VDJ sequences shared by these are shown in **Supplemental Table 8**. Evaluation of cloning frequency showed that the CD4+ CTL1 and CD4+ CTL2 clones experienced larger or medium expansion, the CD4+ Tcm-like and Treg cell clones experienced small or no expansion, and the CD4+ Tra cell clones experience no expansion (**Figure 5C**). Analysis of the transcriptome similarity of these clusters showed that CD4+ T-cell clones with the same receptor sequence had more similar gene-expression profiles than non-clonally expanded T cells (CD4+ CTL2 vs. CD4+ Tra cells, p < 2.2e-16; CD4+ CTL2 vs. Treg cells, p < 2.2e-16; CD4+ Tra vs. Treg cells, p < 2.2e-16; by paired Wilcoxon test), as shown by comparing the Jaccard similarity coefficients for the 200 most abundant genes chosen from each cell type cluster (53) (**Figure 5D**). It is highly possible that CMV-reactivated CD4+ CTL1 and CD4+ CTL2 may be different states of the same group.

## Discussion

Although CD4+ T cells have been shown to play a significant role in anti-CMV immunity, previous methods of measuring CD4+ T cell responses have provided only a partial picture of the involvement of CD4+ T cells in immunological responses to CMV. This study presents a comprehensive profile of CMV pp65-specific CD4+ T cell responses. First, it showed that, of these T cell populations, a surprisingly high percentage (56.68%) consisted of Tregs, with the remaining effector cells being predominantly polyfunctional cells with cytotoxic profiles. Second, this study found that CD4+ CTL2 cells are a more differentiated subset of CD4+ CTL1 cells, evidenced in part by their overlapping TCR repertoires. A key advantage of this study was the use of overlapping pp65 peptide stimulation and CD154 as indicators of CD4+ T cell activation, both of which are independent of MHC haplotype. These results enable further characterization of the CMV-specific CD4+ T cell response and can be compared with responses to other viruses.

CD154 is an effective marker when combined with single-cell mRNA sequencing for high-throughput analysis of virus antigen-specific T cells (25, 27–29). Although traditional research methods based on measurement of secreted cytokines, such as IFNG or TNF, and testing of CMV-specific T cells have proven effective (54–56), they are of limited use when combined with sc-mRNA sequencing due to cell damage caused by intracellular staining. The use of peptide-MHC (pMHC) multimers to isolate antigen-specific T cells based on the specific binding of TCR with pMHC has allowed detailed TCR and phenotypic analysis of single cells (57–59). However, the decreased TCR expression in activated T cells can result in the selection of relatively low antigen-specific T cells bound to tetramer (60), This selection of multimer-binding CD4+ T cells may bias understanding of the phenotype of antigen-specific CD4+ T cells (60). The finding that 83.8% of CMV stimulated but only 17.4% of control CD4+ T cells were positive for *CD154* (*CD40LG*) expression indicates that CD154 is comparable to IFNG and TNF in distinguishing antigen-specific CD4+ T cells.

This study found that the CMV-reactivated Tregs had different inhibitory functions. LAG3 and CTLA4 are classical Treg inhibitory markers, which bind to MHC-II and CD80/CD86, respectively, on other T cells to repress their activation. Perforin/granzyme-induced apoptosis is the main pathway used by cytolytic cells to kill target cells (61, 62), with perforin and granzyme commonly expressed simultaneously. In our study, Tregs were positive for *SRGN*, which encodes a protein involved in maintaining granzyme storage, and highly expressed *GZMB*, but their expression of *PRF1* was limited. These findings suggest that only a few perforin molecules are sufficient to facilitate the entrance of granzyme into target cells, or that granzyme B can induce cell death in a perforin-independent manner (63), by mediating the cleavage of the extracellular matrix to reduce the adhesion of immune cells, inducing their death. These cells also expressed *LGALS1* and *LGALS3*, encoding Gal-1 and Gal-3, respectively, which may also participate in Treg immunosuppressive activity (64). Disruption of Gal-1 was found to attenuate the immunoexpressing effect of Treg cells (65), and Gal-1 from Tregs was observed to induce the dysfunction of effector T cells and modulate their transient calcium influx (66). This regulatory mechanism is not limited to Gal-1 but is also employed by Gal-3 in Tregs (67). Interestingly, this study showed that Tregs expressed CD70, a marker, to our knowledge, commonly expressed on antigen-presenting cells and activated T cells as part of the CD27-CD70 pathway that provides a costimulatory signal. In T cells, CD70 was shown to induce caspase-dependent apoptosis. Although the mechanism by which Tregs exert inhibitory activity may be similar (68), additional studies are needed to determine the function of CD70 in Tregs. Taken together, these findings show that, during CMV infection, the inhibitory activity of Treg cells is not only maintained but reinforced by enhancing cell migration.

The populations of Treg/induced Tregs (iTregs) have been reported to increase during CMV/MCMV latent infection both in humans and in mice (22, 69–72). However, it is not clear whether these increases are due to the expansion of a small population of circulating Forxp3+ nTregs or due to peripheral conversion of antigen-specific CD4+T cells into iTregs. Most of the Tregs in the present study were probably induced from conventional T cells by TGFβ, which is secreted by all CD4 T subsets and maybe by other cell types in PBMC cultures. Moreover, Tregs were found to inhibit immune responses in the spleen but promote virus control in the salivary glands, suggesting that the effects of Tregs are dependent on their location. It is more likely that, in the presence of functional CD4 CTL, the immune system would favor iTregs over newly activated T cells, especially in the peripheral blood, where inflammation can be more harmful than in a relatively restricted tissue.

During acute viral infection, CD4 + T cells assist in the activation of CD8 + T and B cells to clear the virus. During chronic infection, including infections with HCMV, MCMV, herpes simplex virus, varicella zoster virus, murine gammaherpesvirus 68, and Epstein–Barr virus, CD4 + T cells play a direct antiviral role, inhibiting virus lysis and replication. This can result in the establishment of virus latency and prevent disease or death in the host (73–75). It is unclear what mechanisms contribute to the establishment of cytotoxic CD4T in chronic infection. In our study, we found populations of activated CD4 CTLs among large numbers of Tregs. CD4 CTLs induced by latent viruses are independent of co-stimulation, resistant to apoptosis, and less susceptible to suppression by regulatory T cells (Tregs) during repeated antigenic stimulation (76). Interestingly, the number and proportion of CD4 CTL cells expressing immune regulating genes, such as *CTLA-4*, *LAG3*, *IL-2RA*, and *PDCD1*, were at least comparable to, if not greater, than the number and proportion Treg cells. Fewer less resources are therefore available for the activation of other conventional CD4+ T cells. In addition, both CD4 CTLs and Treg cells express IFN-γ and TNF-α, which can promote innate immune responses. Although this study did not determine whether IFN-γ and IFN-expressing Treg cells have enhanced or dampened function, it is likely that the combination of CD4 CTLs and Tregs will result in CD4 CTL dominant immune responses accompanied by increased innate immune responses.

Less is known about bystander activation of CD4+ T cells than of CD8+ T cells, but unrelated memory CD4+ T cells were shown to be activated after repeat tetanus vaccination *via* bystander activation (77), and multiple cytokines sharing a common receptor gamma chain were found to induce CD154/CD40 ligand expression by human CD4+ T lymphocytes *via* a cyclosporin A-resistant pathway (78). We found that CD4+ Tcm-like cells, which exist in an environment containing IFN-γ and IL2, are susceptible to activation by these cytokines. We also found, however, that CMV CD4+ Tcm-like cells showed small clonal expansion, making it difficult to determine whether these CD4+ Tcm-like cells are CMV pp65 antigen-specific.

The present study provides useful information for the characterization of CMV-specific CD4 T cell responses and for comparisons with other virus-specific responses. The method we used to analyze CMV-reactivated CD4+ T cells may be extended to other conditions, such as autoimmune diseases and cancers. Our findings may offer insights into the persistence of CMV and levels of immunopathology. In addition, the detailed information provided in this study, such as cell function and cell interactions, may provide a more nuanced view of CMV-related diseases and allow better design of anti-viral therapies.

## Methods and Materials

### PBMC Preparation

We obtained peripheral blood from three CMV IgG-positive, healthy donors through a research protocol proved by the Beijing Genomics Institution-Shenzhen (BGI-Shenzhen) Institutional Review Board (IRB). PBMCs were immediately isolated from blood collected with an EDTA blood collection tube by density centrifuge method with Histopaque-1077 (Sigma, Cat. 10771) within 2 h, resuspended in 4°C cryopreservation medium consisting of 90% fetal bovine serum (FBS, HyClone, Cat. sh30084.03) and 10% dimethyl sulfoxide (DMSO, Sigma, Cat. D4540), and then placed in Mr. Frosty (Thermo Scientific) in -80°C container. Samples were then moved to liquid nitrogen for long-time storage.

Additionally, 2 ml peripheral blood from each donor was collected using a blood collection tube without any additive, placed at room temperature for 30 min, and centrifuged for 10 min at 2,000g. Then, plasma was collected and heat-shocked for 30 min at 55°C.

### PBMC Stimulation

Frozen PBMC from liquid nitrogen were immediately thawed in 37°C water and resuspended in complete medium (RPMI 1640 medium, 10% NEAA, and 2% autologous plasma; RPMI 1640 and NEAA were purchased from Thermo Fisher with Cat. 72400120 and Cat. 11140050) to a final density of 1*10^7^ per milliliter (ml). We moved 150 μl of cell suspension with three repetitions to each well in the 96-well U-plate (Falcon) and incubated them at 37°C for 2 h. Then, 75 μl culture supernatant in each well was replaced by 75 μl stimulation medium and gently mixed. Cells were cultured in an incubator with 5% CO_2_ at 37°C for 24 h.

The stimulation medium included RPMI 1640 medium (without serum), anti-CD28 (2 μg/ml, Clone G28.5, GeneTex, Cat. GTX14148), and anti-CD40 (2 μg/ml, Clone HB14, Miltenyi, Cat. 130-094-133) with/without CMV peptide (1.2 nmol/ml per peptide). To preserve the surface expression of CD154 on activated T cells, we used anti-CD40 to inhibit the interaction of surface CD154 with its counterpart CD40 as described in the previous study (25). We stimulated PBMCs from three CMV-seropositive donors *in vitro* with CMVpp65 peptides in the presence of anti-CD40 monoclonal antibody, negative control cultured without CMVpp65, and positive control with anti-CD3 and anti-CD28. The CMV pp65 peptide was purchased from Miltenyi (Cat. 130-093-438) and diluted in sterile water.

### Enrichment of CMV pp65-Specific T Cells

Cells were collected and washed with FACS washing buffer (DPBS, 2% FBS, and 1 mM EDTA) for once and resuspended in staining buffer (FACS washing buffer with 10% human plasma and 1% BSA) containing antibodies against CD3, CD4, CD154, and CD69 (**Table 2**). After being incubated on ice for 40 min, cells were washed with FACS washing buffer twice and resuspended in 100 μl washing buffer. The stained cells were analyzed and sorted by a BD FACS Aria II cell sorter (BD Biosciences). For cells stimulated with the CMV peptide, CD3+CD154+ cells were sorted as CMV-specific T cells. For unstimulating cells, monocytes and lymphocytes gated according to the plot of FSC-SSC were sorted respectively and re-mixed as a control. The gating schedule for cell sorting was recorded by BD Aria II, and FACS data were analyzed with FlowJo v10.0.7.

**Table 2:** FACS antibodies.

Antigen	Clone	Fluorophore	Supplier	Dilution
CD3	SK7	FITC	BioLegend	1:100
CD4	RPAT4	PerCP-Cy5.5	eBioscience	1:200
CD154	TRAP-1	PE	BD	1:50
CD69	FN50	BV421	BioLegend	1:50

### Droplet Generation, 10× RNA-Seq, and TCR-Seq Library Preparation and Sequencing

After being counted with C-Chip (inCYTO), CMV-reactivated cells and control cells from all three individuals were mixed separately and diluted with PBS to a final concentration of ~800 cells/μl, and about 20,000 cells per reaction were loaded onto a Chromium Single Cell Chip (10x Genomics). The libraries for RNA-seq and TCR-seq were prepared using the Chromium Single Cell 5′ Library & Gel Bead Kit v2 and Chromium Single Cell V(D)J Human T Cell Enrichment Kit (10x Genomics) following the manufactory’s protocol. Sequences within these libraries were ligated with BGIseq adapters, and then CMV and control libraries were loaded onto the sequencing chip. The RNA-seq libraries were sequenced with an 8-base index read, a 26-base read 1 containing cell-identifying barcodes and unique molecular identifiers (UMIs), and a 100-base read 2 containing transcript sequences on BGIseq500; TCR-seq were sequenced with an 8-base index read, a 150-base read 1 containing cell-identifying barcodes, UMIs and insert starting from the V-gene region, and a 150-base read 2 containing an insert from the C-gene region. The raw data after sequencing were about 10 + 35 Gb per library for RNA-seq and 35 + 35 Gb for TCR-seq.

### Preprocessing Single-Cell RNA-Seq Data

Raw data were split according to sample barcodes into CMV-stimulated (ST) and unstimulated library (CON) and then were filtered, blasted, aligned, and qualified by Cellranger v2.2.0 with reference of refdata-cellranger-GRCh38-1.2.0 for RNA-seq data and Cellranger v3.0.0 with refdata-cellranger-vdj-GRCh38-alts-ensembl-2.0.0 for TCR-seq data. Other parameters were set as default in the software.

### Data Integrating and Cell Clustering

The R package Seurat (79) 3.1.5 was used to integrate and analyze datasets from CMV and control. The merged expression matrix was firstly filtered following the Seurat recommendation (80, 81) and a total of 8,671 cells with unique UMI was obtained. Unsupervised clustering was conducted with Seurat with the parameter res = 0.5.

### Differential Expression Gene Analysis

Differential expression gene (DEG) analysis was conducted by the function *FindMarkers* provided by *Seurat*. To characterize the features of CMV-specific CD4+ T cell response, we used a stricter standard to filter out DEGs between CMV and control CD4+ T cells according to the following standard: for upregulation genes in CMV, adjusted p-value < 0.05, log fold change >1, percentage of cells expressing the gene in the CMV sample (pct.1) >0.8, percentage of cells expressing the gene in control (pct.2) < 0.2; for downregulation genes in CMV, adjusted p-value < 0.05, logFC >1, pct.1 <0.2, pct.2 >0.8.

### Quality Control Metrics and Filtering

CellRanger v2.2.0 software with default settings was used to process the raw FASTQ files, align the sequencing reads to the GRCh38 transcriptome, and generate a filtered UMI expression profile for each droplet.

### Identifying the Sample Identity of Each Droplet

The transcriptome of each donor’s PBMCs was sequenced on the BGI-SEQ500 platform with sequencing type SE200. Raw data with 10 G per sample were obtained. The best-practice workflows recommended by the Genome Analysis Toolkit (GATK) (https://gatk.broadinstitute.org/hc/en-us/articles/360035531192-RNAseq-short-variant-discovery-SNPs-Indels-) were followed to identify single-nucleotide polymorphisms (SNPs) and create VCF files containing the genotype (GT) to assign each barcode to a specific sample. The VCF file and BAM files produced by CellRanger2 were passed to the demuxlet software to deconvolute sample identity (37). The optimal likelihood for the identity of each sample was assigned to the corresponding donor, with each “possible” or “ambiguous” droplet regarded as unclear.

### GO Analysis

To annotate the potential functions of the DEGs of each CD4+ T cell cluster, GO enrichment analysis was performed using the clusterProfiler R package, version 3.14.3 (82), with the differentially expressed feature genes identified by Seurat. The top 20 enriched pathways, ranked by normalized enrichment score, with Franklin Delano Roosevelt (FDR) q-value ≤0. 05 were chosen and visualized.

### Gene Set Enrichment Analysis

Gene set enrichment analysis (GSEA, http://www.broad.mit.edu/gsea) was performed with default sets to determine the cell type of cluster 3. The gene set collection used for GSEA was c7.all.v7.1.symbols.gmt (ftp.broadinstitute.org://pub/gsea/gene_sets/c7.all.v7.1.symbols.gmt).

### TCR Analysis

TCR analyses were performed with the R package scRepertoire and Gliph2. Overlap coefficients were calculated using the intersection of clonotypes divided by the length of the smallest component.

## Data Availability Statement

The data that support the findings of this study have been deposited into the CNGB Sequence Archive of CNGBdb with accession number CNP0001262 (https://db.cngb.org/search/sample/?q=CNP0001262).

## Ethics Statement

The studies involving human participants were reviewed and approved by ethical clearance from the institutional review board of BGI. The patients/participants provided their written informed consent to participate in this study.

## Author Contributions

LT and XL designed this project. MHL and SYW performed experiments together. MHL conduct data analysis. MHL, LT, SYW, KG, LW interpreted the data and drafted the manuscript. LT, MHL, BL, XJZ, MNW revised the manuscript. YL modified the syntax. LT, XL, and BL provided direction. All authors contributed to the article and approved the submitted version.

## Conflict of Interest

ML, SW, KG, LW, XZ, YL, MW and LT were employed by BGI-Shenzhen.

The remaining authors declare that the research was conducted in the absence of any commercial or financial relationships that could be construed as a potential conflict of interest.

## Publisher’s Note

All claims expressed in this article are solely those of the authors and do not necessarily represent those of their affiliated organizations, or those of the publisher, the editors and the reviewers. Any product that may be evaluated in this article, or claim that may be made by its manufacturer, is not guaranteed or endorsed by the publisher.

## Supplementary Material

Supplementary Figure 1 Flow cytometry analysis of cells from the three CMV seropositive donors stimulated with anti-CD3 and anti-CD28 antibodies, stimulated with CMVpp65 peptides, and unstimulated (control). FACS data are missing for unstimulated cells from donor #1. After 24 h, the percentages of T cells expressing CD154 were higher following stimulation with anti-CD3 and anti-CD28 antibodies and with CMV than in the negative control.

Supplementary Figure 2 Distribution of CMV CD4+T cells from each of the three donors. **(A)** UMAP embeddings of CMV CD4+ T cells from each donor. Cells were assigned to each donor using demuxlet (28); ambiguous droplets were regarded as “unclear”. Proportions of cells from each donor are shown on the left. UMAP embeddings were **(A)** colored or **(B)** split by donors. **(C)** Percentage of the five CMV-stimulated CD4+ cell clusters relative to total CD4+ T cells from each donor.
