# Supplementary figures and images for: Dissecting the Landscape of Activated CMV-Stimulated CD4+ T Cells in Humans by Linking Single-Cell RNA-Seq With T-Cell Receptor Sequencing
Source: Front Immunol. 2021 Dec 7;12:779961. doi: 10.3389/fimmu.2021.779961 (PMC8691692; doi:10.3389/fimmu.2021.779961)

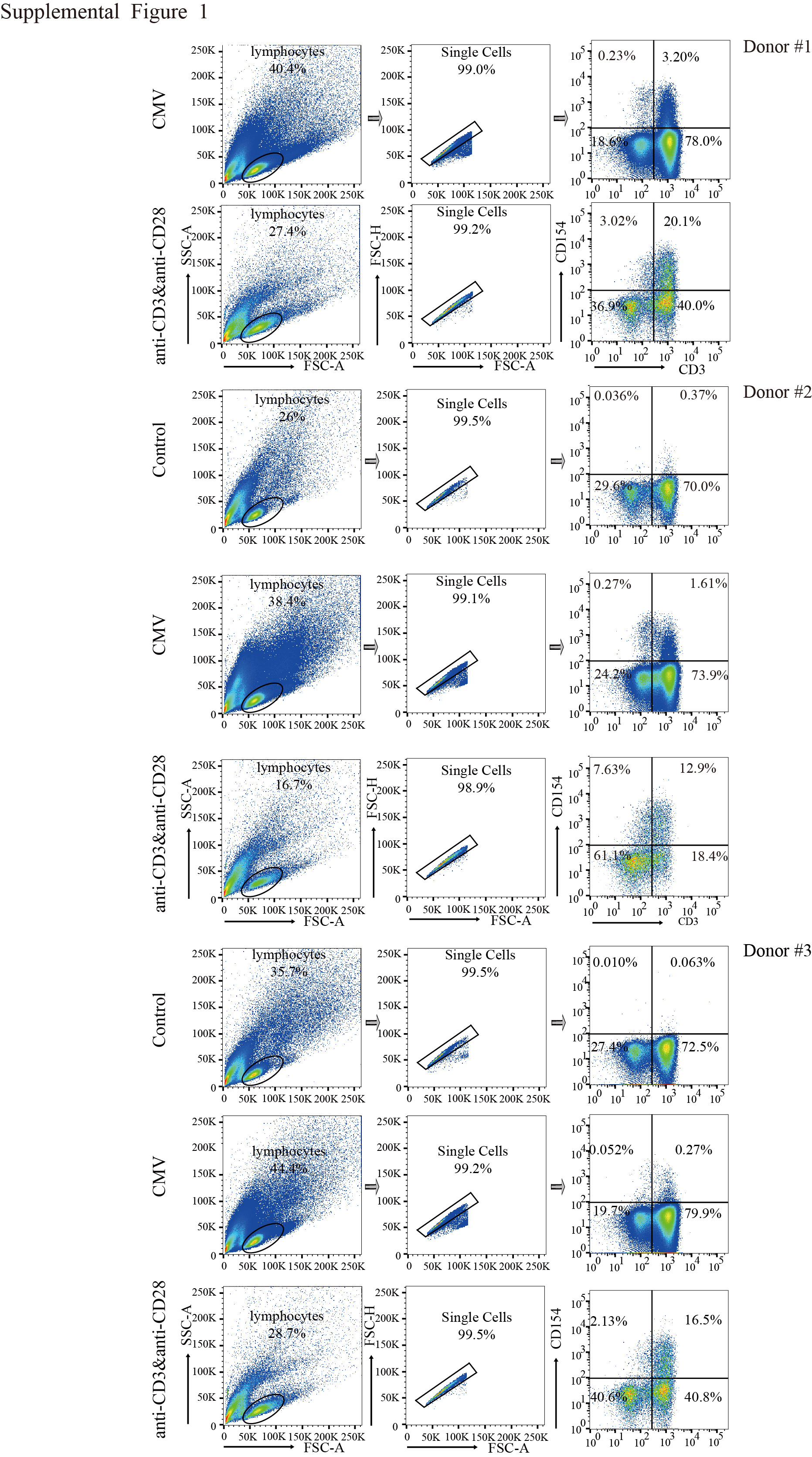

Supplement: Supplementary Figure 1 — Flow cytometry analysis of cells from the three CMV seropositive donors stimulated with anti-CD3 and anti-CD28 antibodies, stimulated with CMVpp65 peptides, and unstimulated (control). FACS data are missing for unstimulated cells from donor #1. After 24 h, the percentages of T cells expressing CD154 were higher following stimulation with anti-CD3 and anti-CD28 antibodies and with CMV than in the negative control. [file Image_1.jpg]

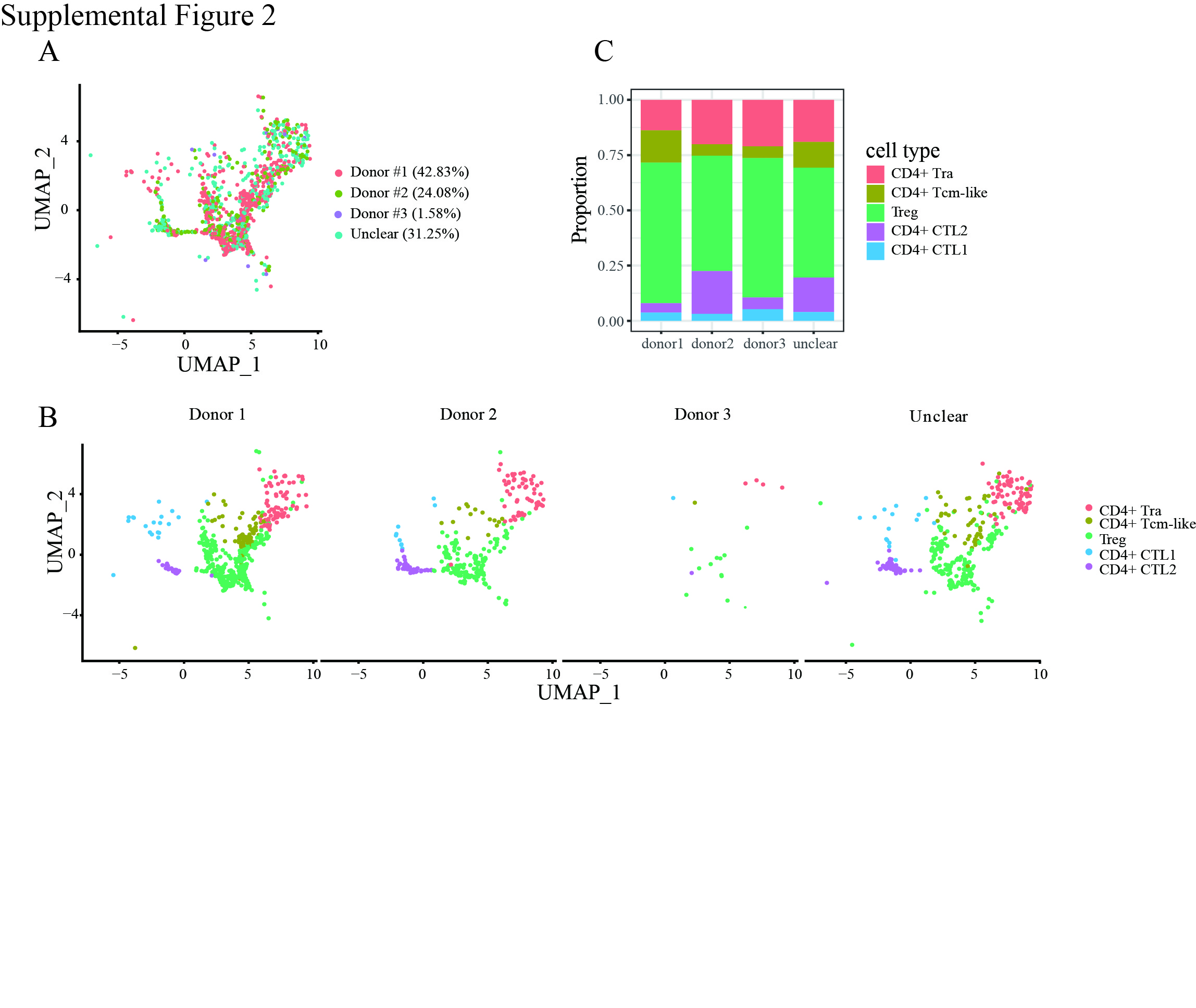

Supplement: Supplementary Figure 2 — Distribution of CMV CD4+T cells from each of the three donors. (A) UMAP embeddings of CMV CD4+ T cells from each donor. Cells were assigned to each donor using demuxlet (28); ambiguous droplets were regarded as “unclear”. Proportions of cells from each donor are shown on the left. UMAP embeddings were (A) colored or (B) split by donors. (C) Percentage of the five CMV-stimulated CD4+ cell clusters relative to total CD4+ T cells from each donor. [file Image_2.jpg]
